# Effects of Different Types and Ratios of Dry Tea Residues on Nutrient Content, *In Vitro* Rumen Fermentation, and the Bacterial Community of Ensiled Sweet Sorghum

**DOI:** 10.3390/microorganisms12112178

**Published:** 2024-10-29

**Authors:** Tong Zhou, Binbin Na, Xingcheng Lei, Yuangan Qian, Yixiao Xie, Yulong Zheng, Qiming Cheng, Ping Li, Chao Chen, Fuyu Yang, Hong Sun

**Affiliations:** College of Animal Science, Guizhou University, Guiyang 550025, China; zlilit@126.com (T.Z.); 15969457062@163.com (B.N.); leixc9972@163.com (X.L.); qianyg183@163.com (Y.Q.); xieyx@gzu.edu.cn (Y.X.); ylzheng3@gzu.edu.cn (Y.Z.); qmcheng@gzu.edu.cn (Q.C.); lip@gzu.edu.cn (P.L.); gzgyxgc3855218@163.com (C.C.)

**Keywords:** tea residues, antioxidant capacity, free amino acids, protein degradation, *in vitro* digestibility

## Abstract

Dry tea residue is a byproduct generated during the production, processing, and storage of tea leaves. The active ingredients and microbial composition of dried tea residue vary depending on different tea processing techniques. This study investigated the effects of six processed dry tea residues—green tea (G), black tea (B), raw Pu’er tea (Z), white tea (W), and ripe Pu’er tea (D)—at two addition ratios (5% and 10%) on the nutritional composition, fermentation quality, *in vitro* fermentation, and bacterial community of sweet sorghum (*Sorghum bicolor*) in the ensiling process. Compared to the control group (CK), the addition of tea residue significantly increased the crude protein (CP) content in silage by 17.9% to 180% (*p* < 0.05), and the content increased with increasing ratios of tea residue. The G10 treatment resulted in the highest CP content, reaching 16.4%. Including tea residue also influenced the ratio of ammonia nitrogen (NH_3_-N) to non-protein nitrogen (NPN). Furthermore, the G and Z treatments at both addition levels increased the total phenolic content, DPPH free-radical scavenging activity, and total antioxidant capacity of the sweet sorghum silage. Except for the Z5 and W10 treatments, the addition of tea residue did not significantly affect *in vitro* dry matter digestibility. Overall, this study showed that incorporating tea residue could enhance the nutritional quality and antioxidant capacity of sweet sorghum silage, and the G5 treatment performed the best. The research results suggested that dried tea residues have potential as silage additives.

## 1. Introduction

Tea is one of the most popular non-alcoholic beverages globally, with an estimated 18 to 20 billion cups consumed daily and over 3 million tons consumed annually [1]. China is a leading producer and consumer of tea, having produced 3.54 million tons in 2023 alone [2]. In China, tea is classified into six categories based on the tea tree variety, leaf-picking season, degree of fermentation, and processing steps. These categories include unfermented green tea, black tea (80–90% fermented), oolong tea (30–60% fermented), fully fermented black tea (100% fermented), white tea (10–20% fermented), and yellow tea (10–20% fermented) [1,3]. Tea contains various active ingredients, including alkaloids, theanine, vitamins, tannins, and polyphenols [4]. The different processing methods used for tea influence the content and form of the active ingredients as well as the microorganisms associated with the tea, thereby imparting unique tastes and flavors. It is these properties of the compounds that enable different types of tea to exert varying effects on human health, such as benefits for gut microbiota regulation [5], antiobesity effects [6], antibacterial activity, and antioxidant properties [7].

The significant global consumption of tea results in the generation of a considerable amount of tea waste. Tea residue is generally considered a byproduct of the processing and preparation of tea and tea beverages. The term “tea residue” encompasses not only the wet tea residues produced during the preparation of tea beverages [8] but also the dry tea residue generated during the production and storage of various types of tea, as well as the accumulation of aged, low-quality tea [9]. India is the second-largest producer of tea in the world, with its factories generating approximately 22.2% of the total national tea yield in annual tea residue production [10]. As the world’s leading tea producer, China’s production of tea residue should not be underestimated. Typically, dry tea residues are burned, discarded, buried, or composted, which often leads to an underappreciation of their potential applications. Therefore, developing strategies to utilize these dried tea leaves to minimize resource waste is a critical concern.

Previous research has predominantly concentrated on the active compounds in tea residues [11], biochar utilization [12], and energy production [13]. Tea residues contain protein, fiber, secondary metabolites, and minerals and are also considered a good animal feed supplement. The 5–8% tannins present in tea leaves and the presence of polyphenol oxidase are retained even after deep processing. These components can inhibit protein degradation by microorganisms during the ensiling process and enhance protein digestibility in ruminants [14]. The presence of tannins in the protein-rich material serves to complex proteins, thereby reducing the extent of protein degradation. Polyphenol oxidase is a copper-containing enzyme that can react with phenolic substrates to produce quinone and modify proteins, forming quinone–protein complexes and inhibiting protein degradation [15]. Tea contains a high crude protein content (22–35%), positioning it as a potential high-protein feed supplement that could enhance the nutritional value of low-protein ensiled forages. Kondo et al. [8] demonstrated that the incorporation of wet tea residues as a silage supplement can elevate the protein content of silage; specifically, the addition of 20% wet green tea residues increased the protein content of silage oats from 5.6% to 8.14%, which in turn improved animal performance and bolstered immunity [16]. The inclusion of an appropriate quantity of tea residues in ruminant diets has the potential to increase feed intake [17], diminish methane emissions, and alter rumen fermentation and the composition of the gut microbiota [18]. Moreover, tea residues contain a significant number of bioactive components, such as tea polyphenols, which exhibit strong antioxidant activity [1]. The application of tea residues as feed additives could supplement deficient nutrients in animal diets, lower the incidence of disease in ruminants, and reduce economic costs, resulting in economic and environmental benefits. Although some studies have investigated tea residues as silage additives to reduce protein degradation during the ensiling process [8,16], these investigations have primarily focused on the effects of 1–2 types of wet tea residues. In contrast, the dry residue resulting from deep processing, which is discarded tea of substandard quality, contains a higher content of protein and active compounds. It is currently unknown how their use as silage additives affects the silage quality of forage.

Sweet sorghum (*Sorghum bicolor*) is considered a promising energy crop and silage material due to its strong adaptability to various environmental conditions, short maturation period, and high content of water-soluble carbohydrates (WSCs), which is approximately 14% [19]. However, the high sugar content and moisture content, combined with the low protein content, make sweet sorghum susceptible to nutrient loss and mold growth during silage harvesting [20]. This research employed tea residues derived from six distinct processing methods—green tea (G), black tea (B), raw Pu’er tea (Z), white tea (W), and ripe Pu’er tea (D)—as additives in silage and set two addition ratios (5% and 10%) to investigate their impact on the nutritional composition, fermentation quality, microbial community structure, and *in vitro* digestibility of sweet sorghum to provide a reference for the efficient utilization of dried tea residues.

## 2. Materials and Methods

### 2.1. Material Preparation

Sweet sorghum was manually cultivated and harvested from Guanling Buyi and Miao Autonomous County (25°56′ N, 105°37′ E, altitude 1121 m), Guizhou, China. The harvested sweet sorghum was chopped to 4–5 cm and treated with the following: no addition (CK), G, B, Z, W, and D. All the tea residue treatments were applied at ratios of 5% (5) and 10% (10) (fresh matter of sweet sorghum). All tea residues used in this study were provided by Guizhou Dachacang Tea Tourism Ecological Agriculture Development Co., Ltd. (Huishui, China). Every treatment was homogeneously mixed and packaged in polyethylene plastic bags (300 g per bag) and vacuumed tightly. After 90 days of ensiling, 44 samples (11 treatments × 4 replicates) were opened to determine chemical composition, antioxidant activity, microbial population, and bacterial community composition. Samples were kept at ambient temperature (0–26 °C).

### 2.2. Chemical Composition and Fermentation Quality Analysis

The content of dry matter (DM) and crude protein (CP) was measured using a method previously described by Helreich [21]. Neutral detergent fiber (NDF) and acid detergent fiber (ADF) content were measured using a method previously described by Van Soest et al. [22]. The water-soluble carbohydrate (WSC) content was analyzed using the anthrone–sulfuric acid method [23], and the nonprotein nitrogen (NPN) content was determined using the Kjeldahl method after precipitation with trichloroacetic acid. The peptide nitrogen (Peptide-N) content in the sample was determined according to the formula: Peptide-N = NPN − (FAA-N + NH_3_-N) [24].

Each sample (10 g) was mixed with 90 mL sterile water and stored at 4 °C overnight. The pH was determined using a pH meter (Rex PHS-3E, INESA Scientific Instrument Co., Ltd., Shanghai, China). The ammonia-N (NH_3_-N) content was determined according to the sodium hypochlorite and phenol method [25]. The lactic acid (LA), acetic acid (AA), propionic acid (PA), and butyric acid (BA) concentrations were measured using high-performance liquid chromatography (HPLC) according to the methods outlined in [26], and the free amino acid nitrogen (FAA-N) content was measured using the ninhydrin hydrazine sulfate colorimetric method.

### 2.3. Total Phenolic Content

The total phenolic (TP) content was determined using the methods described by Zhang and Liu [27]. Briefly, the tea polyphenol extract, 7.5% sodium carbonate, and Folin–Ciocalteu reagent (Shanghai Macklin Biochemical Technology Co., Ltd., Shanghai, China) were combined, and the mixture was left to react for 30 min at room temperature. A UV spectrophotometer (SP-1920 UV Visible Spectrophotometer, Shanghai Spectrum Instruments Co., Ltd., Shanghai, China) was used to measure the absorbance at 730 nm, and the gallic acid standard curve equation was used to determine the phenolic chemicals present in the sample.

### 2.4. Analysis of Free Amino Acids

Preparation of free amino acid samples: the sample (2.0 g) was placed in a 250 mL conical flask along with 200 mL of boiling water, heated in a water bath for 10 min, and then cooled to room temperature. The supernatant (2 mL) was filtered through a 0.22 μm syringe filter membrane [28]. The amounts of 8 free amino acids in the samples were measured using an automated amino acid analyzer (Sykam S-433, Eresing, Germany) [29].

### 2.5. Bacterial Community Analysis

#### 2.5.1. Sequencing DNA Extraction and PCR

Following the methods of Xie et al. [30], 10 g of sample was taken, mixed with 90 mL of sterile physiological saline (8.5 g/L NaCl), sealed, and shaken on a shaker at 120 r/min for 2 h. The sample was filtered and collected using centrifugation at 12,000× *g* for 15 min, after which the total DNA of the mixed sample microbial community was extracted using a PowerSoil DNA Isolation Kit (MO BIO Laboratory, Carlsbad, CA, USA). Primers in the V3~V4 regions of the bacterial 16S rRNA gene were used to sequence and amplify the obtained DNA template. The PCR mixture was 20 μL and included 10 μL of Premium Ex Taq, 0.5 calibrated template DNA, 0.4 calibrated forward primer F336, and 0.4 reverse primer R806 for L. The mixture was subsequently supplemented with sterilized ddH_2_O to 20 μL. Predenaturation was performed at 95 °C for 5 min, denaturation at 94 °C for 30 s, annealing at 57 °C for 30 s, extension at 72 °C for 90 s, a final reaction cycle number of 35, and extension at 72 °C for 10 min. The PCR products were detected using 2% agarose gel electrophoresis and sequenced on a computer. The Illumina MiSeq sequencing platform (Illumina Corporation, San Diego, CA, USA) was used for sequencing by Novo Microbiology Co., Ltd. (Sacramento, CA, USA).

#### 2.5.2. Data Analysis

After obtaining the final valid sequence by removing low-quality reads and chimeric sequences, sequence analysis was performed with Uparse software (Uparse v7.0.1001). Sequences with ≥97% similarity were assigned to the same operational taxonomic units (OTUs). The representative sequence for each OTU was screened for further annotation. For each representative sequence, the Silva Database (http://www.arb-silva.de/) was used to annotate taxonomic information [31]. In order to study the phylogenetic relationships among different OTUs and the differences in dominant species in different samples (groups), multiple sequence alignment was conducted using MUSCLE software (Version 3.8.31). OTU abundance information was normalized using a standard sequence number corresponding to the sample with the least sequences.

### 2.6. In Vitro Rumen Fermentation

The samples of materials and silages were incubated *in vitro* with rumen fluid in calibrated glass syringes [32]. Each sample (200 mg) was weighed into a 100 mL calibrated glass syringe (Haberle Labortechnik, Lonsee, Germany). A buffered mineral solution was prepared, and 20 mL of the solution was distributed to the incubation bottles in a water bath. Fresh rumen fluid was collected from Simmental cattle of Guizhou Junong Meat Industry Development Co., Ltd. (Guiyang, China), using a thermos bottle in the early morning, which was then immediately transferred to the laboratory, filtered through four layers of cheesecloth, and flushed with CO_2_. Rumen fluid (10 mL) was combined with the prepared buffered mineral solution, which was maintained in a water bath at 39 °C. All handling was performed under continuous blanket flushing with CO_2_. The glass syringes containing the samples and rumen fluid–buffer mixtures were incubated in an incubator at 39 °C, and gas production was subsequently measured before incubation (0 h) and at 3 h, 6 h, 9 h, 12 h, 18 h, 24 h, 36 h, 48 h, and 72 h after incubation.

### 2.7. Statistical Analysis

The data were organized using Microsoft Excel 2019, and two-way analysis of variance (ANOVA) was performed using SPSS 26.0 (IBM Corp.) software. Duncan’s test was used for multiple comparisons, and *p* < 0.05 indicated statistical significance.

## 3. Results

### 3.1. Chemical Composition of the Raw Materials

As shown in Table 1, the fresh sweet sorghum has a DM content of 28.0%, and the CP, WSC, NDF, and ADF contents were 5.76%, 15.5%, 55.7%, and 36.3%, respectively. In addition, the CP and NPN contents in the different types of tea were greater than those in fresh sweet sorghum, with G having the highest CP (33.8%) and NPN contents (9.06%). All the tea residues had lower NDF and ADF contents than the fresh forage.

### 3.2. Fermentation Quality and Nutrient Composition in Sweet Sorghum Silage with the Addition of Tea Residues

Compared with the CK group, except for the G5 and D10 treatments, the pH values of all the tea residue treatments were lower than that of the control and were below 4.5 (Table S1). The LA concentrations of all the tea residue types were higher except for those in the G5 and B (5, 10) treatments (*p* < 0.05). Among tea residue types, there are different effects on improving fermentation quality. The concentrations of LA and AA in the G5, Z (5, 10), W5, and D5 treatments were higher than those in the other treatments, resulting in a lower pH (*p* < 0.05). The chemical composition of the silage is shown in Table 2. Compared with those of the CK group, all the tea residue treatments improved the CP content of the sweet sorghum silage; these parameters significantly improved with increasing tea residue addition ratio (*p* < 0.05), and the highest CP content was in the G10 treatment, which was 180% greater than that in the CK group. Compared with the CK group, all tea residue treatments increased the TPN content in sweet sorghum silage by over 44% (Table 3), while reducing the NPN content. The G10 and B (5, 10) treatments resulted in the lowest NPN contents, which decreased by 48.7%, 53.7%, and 49.2%, respectively, compared to those in the CK group. In addition, regardless of the types and ratios of tea residue added to the treatment, the NH_3_-N content in the silages decreased (*p* < 0.05), with the lowest NH_3_-N contents occurring in the B10 and Z10 treatments, which were 70.03% and 75.5% lower, respectively, than those in the control.

### 3.3. Total Phenolics and Antioxidant Capacity in Sweet Sorghum Silage with the Addition of Tea Residues

**Figure 1:**
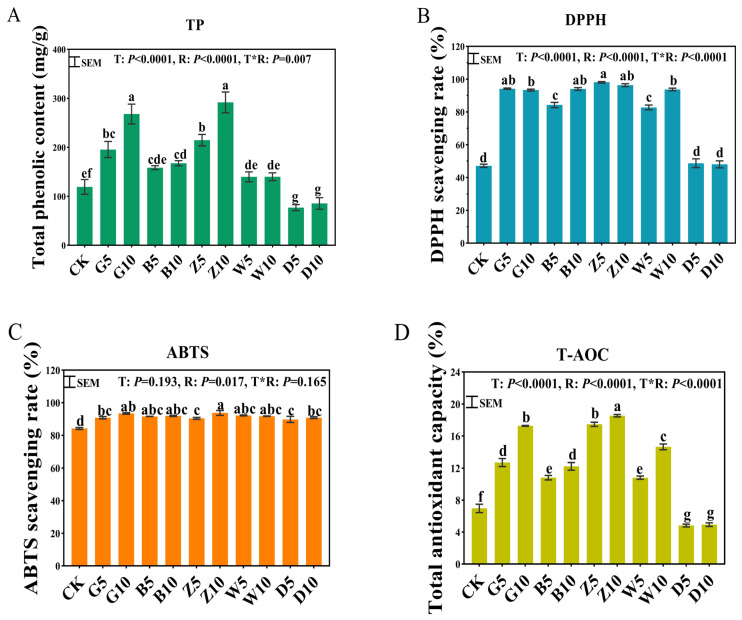
Total phenolic content and antioxidant activity in sweet sorghum silage with the addition of tea residues. (**A**) Tea polyphenol content; (**B**) DPPH radical scavenging rate; (**C**) ABTS radical scavenging rate; (**D**) total antioxidant capacity. The different lowercase letters (a–g) above the column indicate significant differences among treatments (*p* < 0.05); T, treatment; R, additive ratio; T*R, the interaction between the addition ratio and the type of tea residue added.

The effect of adding different types of tea residue on the total phenolic content significantly differed (*p* < 0.05), and the TP content increased with increasing tea addition ratio (Figure 1). Compared with the CK group, except for the D group, which had a decreased total phenolic content, all the other treatments significantly increased the TP content of fermented sweet sorghum (*p* < 0.05). Although the amount of LAB processed was above the 5 lg cfu/g level of long-term anaerobic fermentation preservation (Table S2), the number of LAB in the G10 and D (5, 10) treatments was significantly higher than other treatments. At the same ratio, the G5, B10, and Z (5, 10) treatments showed higher radical scavenging ability (*p* < 0.05).

### 3.4. Free Amino Acids in Sweet Sorghum Silage with the Addition of Tea Residues

**Figure 2:**
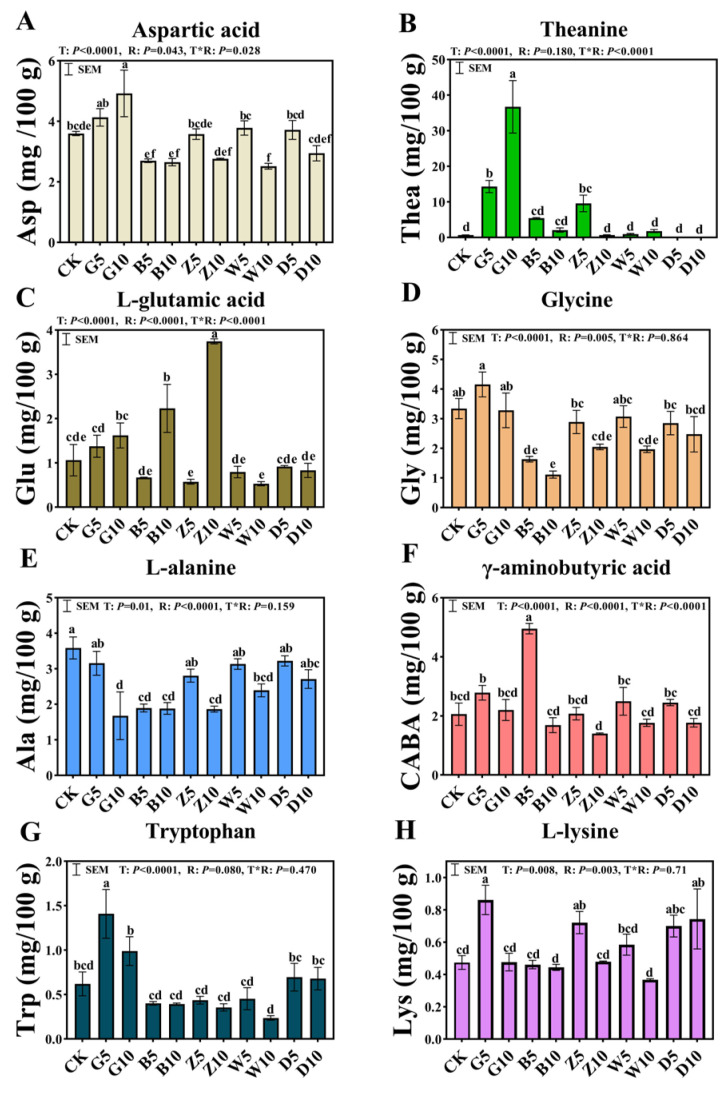
Contents of 8 free amino acids in sweet sorghum silage with the addition of tea residues. (**A**) Aspartic acid; (**B**) theanine; (**C**) L-glutamic acid; (**D**) glycine; (**E**) L-alanine; (**F**) γ-aminobutyric acid; (**G**) tryptophan; (**H**) L-lysine. The different lowercase letters (a–f) above the column indicate significant differences among treatments (*p* < 0.05); T, treatment; R, additive ratio; T*R, the interaction between the addition ratio and the type of tea residue added.

The concentrations of eight free amino acids (Figure 2) as well as the type and ratio of tea residue had significantly interactive effects on the levels of aspartic acid, theanine, glutamic acid, and γ-aminobutyric acid (*p* < 0.05). Each treatment significantly influenced the concentrations of glycine, alanine, tryptophan, and lysine (*p* < 0.05), with concentrations decreasing as tea residue was added. Except for alanine (Ala), the amino acid concentrations in the G5 treatment were higher than those in the CK group.

### 3.5. Bacterial Community Composition in Sweet Sorghum Silage with the Addition of Tea Residues

**Figure 3:**
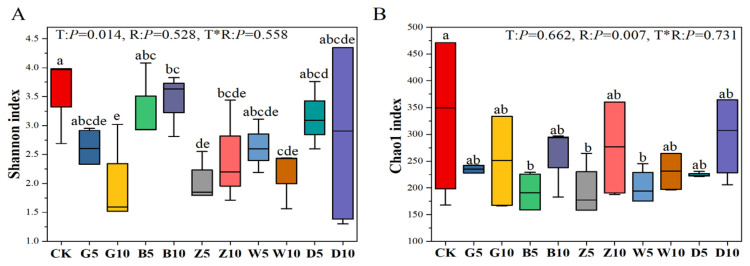
Alpha diversity in sweet sorghum silage with the addition of tea residues. (**A**) Shannon index; (**B**) Chao1 index. The different lowercase letters (a–e) above the column indicate significant differences among treatments (*p* < 0.05); T, treatment; R, additive ratio; T*R, the interaction between the addition ratio and the type of tea residue added.

After anaerobic fermentation, except for the D10 treatment, all the other tea treatments reduced the number of features in ensilaged sweet sorghum. The coverage index in the table was greater than 0.99 (Table S3). Overall, the Shannon and Chao 1 indices of all the tea residue addition treatments were lower than those of the control group (Figure 3). To analyze the distribution and structure of bacterial communities in sweet sorghum silage with varying ratios and types of tea residue, a PCoA analysis based on the Bray–Curtis distance was conducted (Figure S1). The results of the PCoA analysis revealed significant separation among the different types of tea residues added, indicating that the type of tea residue is associated with changes in the microbial community of sweet sorghum silage.

**Figure 4:**
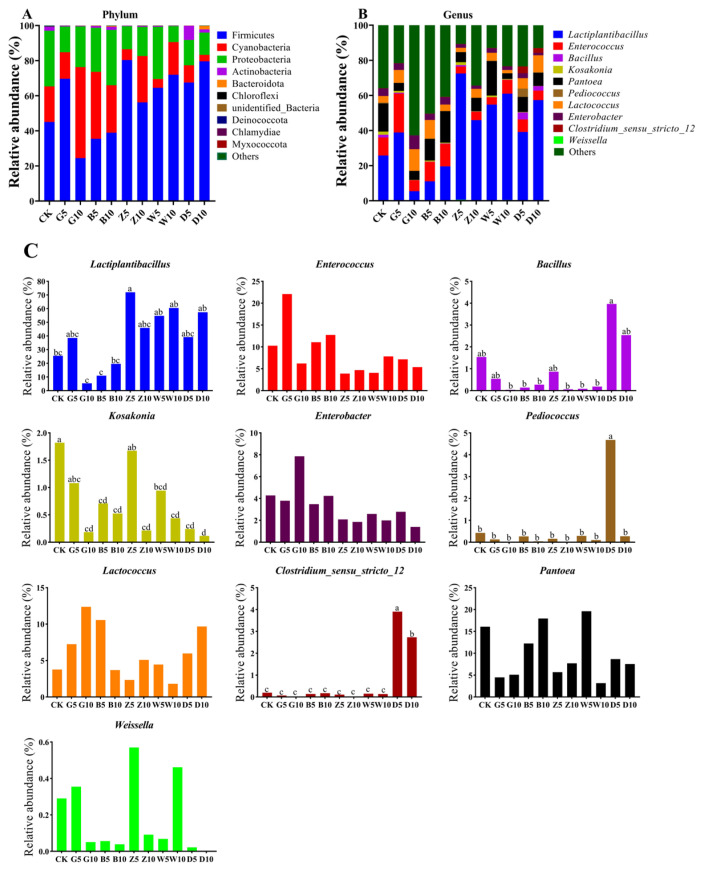
Relative abundance of the silage bacterial composition. (**A**) Relative abundance of bacteria at the phylum level; (**B**) genus level; (**C**) relative abundance of bacteria at the top 10 genus level. The different lowercase letters (a–d) above the column indicate significant differences among treatments (*p* < 0.05).

The changes in the microbial community in sweet sorghum silage with different tea addition types and ratios are shown in Figure 4. Compared with the CK group, the G10, B (5, 10), Z10, and W10 treatments increased the relative abundance of Cyanobacteria in silage, whereas the D (5, 10) treatments both reduced Cyanobacteria in silage. The dominant genera involved in the CK treatment were *Lactiplantibacillus* (25.5%), *Enterococcus* (10.3%), *Pantoea* (16.1%), and *Lactococcus* (3.7%). However, the relative abundance of the dominant bacteria in sweet sorghum silage shifted from *Enterococcus*, *Pantoea*, and others to *Lactiplantibacillus* in the tea treatments. Especially in the Z5 treatment, the relative abundance of *Lactiplantibacillus* was 71.9%, which was the highest of all the treatments.

**Figure 5:**
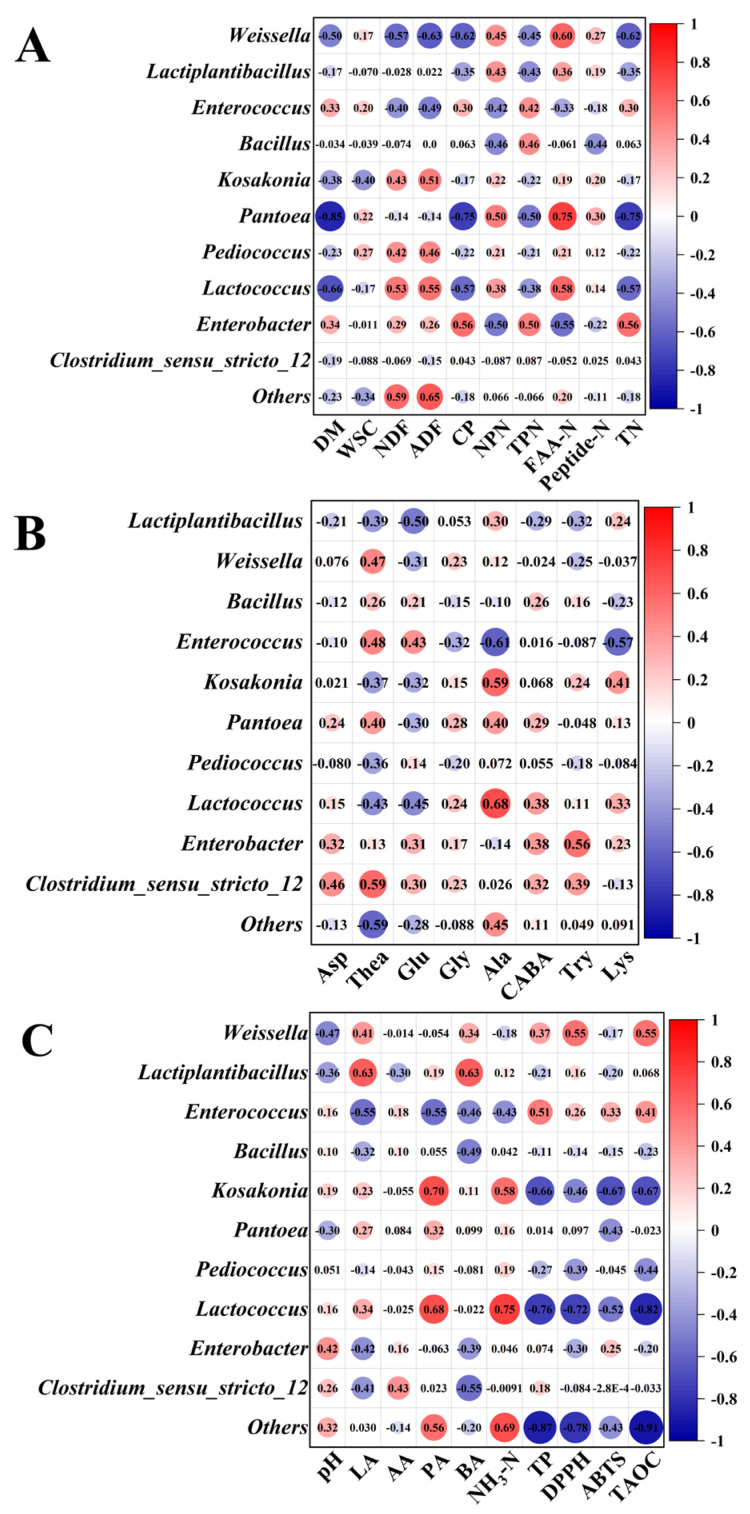
Spearman correlations between the bacterial community and (**A**) chemical composition; (**B**) fermentation characteristics; and (**C**) free amino acids.

The Spearman correlation of the genus level and chemical composition are shown in Figure 5. *Lactiplantibacillus* was positively correlated with LA and BA concentrations and negatively correlated with TP, theanine, glutamic acid, and tryptophan (*p* < 0.05). *Lactococcus* was positively correlated with LA, AA, and PA concentrations and negatively correlated with TP and antioxidant capacity (*p* < 0.05). *Enterococcus* was negatively correlated with the contents of NDF, ADF, NPN, LA, NH_3_-N, alanine and lysine (*p* < 0.05), whereas the contents of TP, theanine, and glutamic acid were positively correlated (*p* < 0.05).

### 3.6. In Vitro Rumen Fermentation in Sweet Sorghum Silage with the Addition of Tea Residues

**Figure 6:**
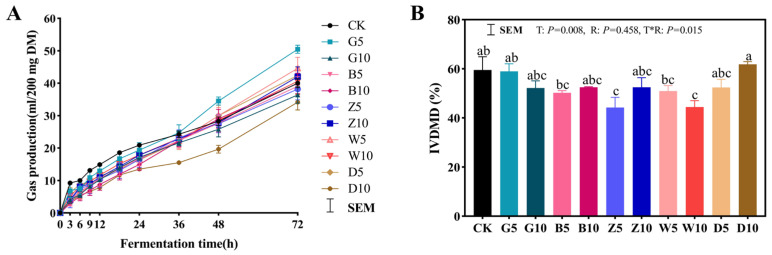
*In vitro* rumen fermentation in sweet sorghum silage with the addition of tea residues. (**A**) Gas production and (**B**) the digestibility from sweet sorghum silage fermented *in vitro* for 72 h. The different lowercase letters (a–c) above the column indicate significant differences among treatments (*p* < 0.05); T, treatment; R, additive ratio; T*R, the interaction between the addition ratio and the type of tea residue added.

After 72 h of *in vitro* rumen fermentation, the gas production of each group was stable in this study (Table 4 and Figure 6A). The theoretical gas production and gas production rates decreased as the ratio increased in the different types of tea residue treatments. The maximum and minimum theoretical gas productions were observed under the G5 and D10 treatments, respectively. Furthermore, the G5 treatment had a significantly higher CH_4_ yield than the other treatments (*p* < 0.05), while there were no significant differences between the other treatments and the CK group. After 72 h of *in vitro* fermentation, compared with those of the CK group, the Z5 and W10 treatments reduced *in vitro* dry matter digestibility (IVDMD) (Figure 6B), whereas the G (5, 10), B (5, 10), Z10, W5, and D10 treatments did not affect IVDMD.

## 4. Discussion

Generally, the WSC content is a limiting factor for ensiling, and the WSC content of fresh sweet sorghum (15.5%) was greater than the theoretical requirement (5.0%) in high-quality fermentation [33]. This means that microorganisms, especially LAB, had enough utilized WSC content as the substrate for faster growth while producing secondary metabolite organic acids to inhibit the growth of other various bacteria. The nutritional composition of tea from different processing technologies and fresh sweet sorghum varies significantly. Among the different types of tea residues, the chemical composition could be consumed by microorganisms during oxidation, microbial fermentation, and other enzymes [34], resulting in a decrease in content.

The pH during silage is an important factor in the accumulation of organic acids because it affects the composition and structure of bacterial communities, metabolic pathways, and enzyme activity [35]. Among tea residue types, the concentrations of LA and AA in the G5, Z (5, 10), W5, and D5 treatments were higher than those in the other treatments, resulting in a lower pH. LA produced by LAB is usually the most concentrated acid in silage, approximately 10 to 12 times stronger than any other major acid [36]. This is why the G5, Z (5, 10), W5, and D5 treatments have lower pH.

All the tea residue treatments improved the CP content of the sweet sorghum silage; this can be attributed to the high CP content (20–30%) contained in the tea residues. This is consistent with Kondo et al. [8], who found that adding wet tea residue can improve the CP content of silage. Therefore, adding tea residue to forage can effectively increase the CP content, which was consistent with the results reported by Guo et al. [37], who reported that tea residue additives can serve as protein supplements.

Generally, protein degradation in silage can be roughly divided into two stages. The first stage involves the hydrolysis of plant peptides to form free amino acids and peptides, which are subsequently degraded by microbial activity into various products [38]. Due to the deamination of amino acids by microbial activity, proteins in forage are degraded into NPN and nondigestible NH_3_-N forms and reduce the utilization of nitrogen by livestock. Therefore, NPN and NH_3_-N are important indicators of protein degradation. The addition of tea residue in this study may reduce the degree of protein hydrolysis due to the rapid decrease in pH during anaerobic fermentation inhibiting the degradation of proteins by *Clostridium*, *Enterobacterium*, *Bacillus*, and *Enterococcus*, as well as the activity of proteolytic enzymes in plants [36]. In addition, the compounds in tea may reduce protein hydrolysis. Similar research has shown that substances such as tannins and polyphenol oxidase can be combined with protein to reduce proteolysis during anaerobic fermentation [8,39], and substances can inhibit deamination and reduce proteolysis during anaerobic fermentation by inhibiting the activity of ammonia-producing microorganisms [1]. Based on the different fermentation characteristics of different types of tea, adjusting the addition ratio and type can regulate microorganisms through various mechanisms to improve the quality of anaerobic fermentation.

Excessive radicals produced by protein oxidation, lipid oxidation, and sugar oxidation during microbial metabolism can lead to oxidative stress and become pathogenic factors [40]. Phenolic compounds are natural antioxidants, and tea polyphenols with high levels of active hydroxyl groups, such as epigallocatechin gallate, exhibit higher antioxidant activity than those with fewer hydroxyl groups. Green tea and raw Pu’er tea with similar processing techniques have relatively high levels of polyphenols and epigallocatechin gallate; therefore, G treatment results in a relatively high antioxidant capacity. Similarly, extracts rich in phenolic acids, flavonoids, and flavonoid glycosides have been shown to have significant antioxidant and free-radical scavenging activities. In addition, polyphenols reduce the production of inflammatory mediators by regulating enzyme activity related to oxidative stress in metabolic pathways [40,41]. The antioxidant activity provided by the tea treatments may improve animal performance and meat quality [42].

The composition of free amino acids in silage can impact its palatability and overall quality. Some amino acids, including aspartic acid, glutamic acid, and theanine are responsible for the umami taste [43]. Each treatment significantly influenced the concentrations of glycine, alanine, tryptophan, and lysine (*p* < 0.05), with concentrations decreasing as tea residue was added. The variation in amino acid content observed in relation to the ratio of tea residues may be attributed to the relative activity of microorganisms and plant proteases. An increase in their content can enhance the palatability of silage. Theanine is a distinctive amino acid present in tea, and it is also the most prevalent amino acid in tea. It is a unique non-protein amino acid that exerts sedative and anti-inflammatory effects [29]. The ensiling process also results in significant degradation of amino acids. Free amino acids undergo a series of chemical reactions, including transamination, dehydrogenation, decarboxylation, and reduction, which result in the formation of acids, alcohols, aldehydes, esters, and other compounds [44]. The majority of the protein in the tea residue treatment in this study was in the form of TPN, which means that the inclusion of tea residue was beneficial for reducing protein hydrolysis and amino acid degradation.

The microbial community richness indices in silage were evaluated using the Shannon and Chao1 indices. The Shannon and Chao 1 indices of all the tea residue addition treatments were lower than those of the control group. The relative abundance of previously complex microbial varieties rapidly decreases during the ensiling process as the number of LAB increases and the pH decreases. This finding is consistent with the findings of Ren et al. [45], who reported that low pH could inhibit the growth of bacteria and reduce the Shannon and Chao 1 indices. Additionally, reports have found that adding other flavonoid-rich residues (wormwood) to alfalfa silage reduces the Shannon index [46]. Moreover, as the addition ratio increased, the Shannon and Chao 1 indices decreased among the same tea-type treatments. Tea residue greatly reduced the diversity and evenness of bacterial communities in sweet sorghum silage, and the greater the ratio of tea residue added was, the lower the diversity and richness of bacteria in sweet sorghum silage. We speculate that during silage, the active ingredients in tea may reduce bacterial diversity by increasing the abundance of dominant bacteria and inhibiting miscellaneous bacteria.

Generally, Firmicutes and Proteobacteria typically replace Cyanobacteria to attach to tea to variable degrees during the processing of tea residues due to differences in the processing technologies utilized [47]. The G10, B (5, 10), Z10, and W10 treatments increased the relative abundance of Cyanobacteria in silage compared to the CK group, whereas the D (5, 10) treatments both reduced Cyanobacteria in silage. Since ripe Pu’er tea is a post-fermented tea, it takes longer to ferment, which could account for why the D (5, 10) treatments reduced the relative abundance of Cyanobacteria. This finding was consistent with the results of Xue et al. [48], who reported that the dominant phylum in ripe Pu’er tea was Firmicutes, whereas Cyanobacteria was the dominant phylum in raw Pu’er tea. Furthermore, Cyanobacteria are commonly found in the soil, water, and phyllosphere [49]; therefore, we speculated that soil dust exposed to the air will be doped into tea during the drying process, increasing the relative abundance of Cyanobacteria. LAB were the most abundant members of Firmicutes and were also the dominant bacteria in the silage. However, the relative abundance of *Lactiplantibacillus* was lower in the G10 and B (5, 10) treatments compared to that in the CK group in this study, which could partly explain the lower concentration of LA in the G10 and B (5, 10) treatments than in the CK group. There was no significant correlation between *Lactiplantibacillus* and the total phenolic content. This finding was consistent with those of Lin et al. [50] and Wang et al. [51] who reported that polyphenols did not affect *Lactobacillus*. Therefore, other unique substances present in the G and B groups may affect the activity of *Lactiplantibacillus*. The negative correlation between total phenolics and *Lactococcus* is different from the research results and requires further research to explain.

In general, *in vitro* gas production is highly dependent on the availability of fermentable carbohydrates and nitrogen. Higher gas production is observed when the degradability of rumen feed increases [52]. The maximum and minimum theoretical gas productions were observed under the G5 and D10 treatments, respectively. This was consistent with Ramdani et al. [53], who found that the *in vitro* gas production of green tea was higher than that of black tea. Furthermore, the G5 treatment had a significantly higher CH_4_ yield than the other treatments, while there were no significant differences between the other treatments and the CK group. This might be due to the tannins in the sweet sorghum and tea residue decreasing the activity of H_2_ transfer between species of methanogenic bacteria in the rumen, which in turn reduces the production of CH_4_. For a ruminant, most of the DM and fiber in forage or the diet are ruminal digested [54]. Therefore, *in vitro* dry matter digestibility is an appropriate indicator reflecting the quality of silage [38]. Previous studies showed that tannins and phenolic compounds in tea inhibit the growth of certain rumen bacteria and rumen protozoa and reduce DM digestibility [54,55], whereas the G (5, 10), B (5, 10), Z10, W5, and D10 treatments did not affect IVDMD, possibly as these treatments did not inhibit microbial metabolism.

## 5. Conclusions

In conclusion, this study showed that the addition of different types of tea residues substantially enhances the fermentation quality of ensiled sweet sorghum. Compared with the other treatments, the G and B treatment groups contribute to an increase in CP and TP-N contents while decreasing NH_3_-N and FAA-N contents in NPN, and did not affect IVDMD, with G5 having a more positive effect on improving fermentation quality and inhibiting protein degradation than other treatments. These results will provide a theoretical foundation for utilizing dried tea residue to reduce protein degradation and increase antioxidant capacity.

## Figures and Tables

**Table 1:** Chemical compositions of fresh forage and raw tea materials.

Items	Fresh Forage	Tea Residue Materials				
		G	B	W	Z	D
Dry Matter (% FM)	28.0	-	-	-	-	-
Crude Protein (% DM)	5.76	33.8	29.8	14.5	27.2	30.9
Water Soluble Carbohydrate (% DM)	15.5	4.18	7.80	8.49	4.47	0.44
Nonprotein-N (% TN)	0.29	9.06	7.65	4.05	8.17	6.51
Neutral Detergent Fiber (% DM)	55.7	33.6	33.8	33.9	26.0	44.8
Acid Detergent Fiber (% DM)	36.3	8.13	14.0	18.6	7.74	36.8
DPPH Scavenging Rate (%)	50.4	91.5	95.3	94.1	91.1	43.4
ABTS Scavenging Rate (%)	89.9	92.5	99.3	90.4	92.3	91.4
Total Antioxidant Capacity (%)	5.60	18.8	18.3	17.6	19.3	4.47

DM, dry matter; FM, fresh matter; TN, total nitrogen.

**Table 2:** Chemical composition of tea residues with different addition ratios and types.

Treatment (T)	Ratio (R)	DM (% FM)	CP (% DM)	WSCs (% DM)	NDF (% DM)	ADF (% DM)
CK	0%	22.1 ^e^	5.86 ^h^	3.36 ^de^	47.8 ^abc^	26.5 ^bc^
G	5%	25.7 ^cd^	11.1 ^d^	3.98 ^cd^	46.7 ^abc^	27.3 ^b^
	10%	30.3 ^a^	16.4 ^a^	3.64 ^de^	44.9 ^cd^	24.3 ^cd^
B	5%	26.7 ^c^	10.7 ^d^	6.22 ^a^	45.6 ^bcd^	26.4 ^bc^
	10%	29.7 ^a^	14.0 ^bc^	4.41 ^bc^	47.2 ^abc^	28.0 ^b^
Z	5%	24.7 ^d^	8.94 ^e^	4.80 ^bc^	46.4 ^abcd^	26.0 ^bc^
	10%	29.8 ^a^	13.4 ^c^	5.06 ^de^	45.1 ^cd^	25.6 ^bcd^
W	5%	25.2 ^d^	6.91 ^g^	4.53 ^b^	49.1 ^abc^	28.2 ^b^
	10%	28.4 ^b^	7.87 ^f^	3.28 ^b^	40.7 ^d^	23.2 ^d^
D	5%	25.7 ^cd^	10.9 ^d^	3.35 ^de^	51.9 ^a^	32.2 ^a^
	10%	29.6 ^a^	14.4 ^b^	3.15 ^e^	51.6 ^ab^	32.3 ^a^
SEM		0.557	0.293	0.355	2.647	1.155
*p*-value	T	0.009	<0.001	<0.001	<0.001	<0.001
	R	<0.001	<0.001	<0.001	0.091	0.016
	T*R	0.06	<0.001	0.002	0.108	0.002

The different lowercase letters (a–h) above the column indicate significant differences among treatments (*p* < 0.05). CK, no addition; G, green tea; B, black tea; Z, raw Pu’er tea; W, white tea; DM, dry matter; D, ripe Pu’er tea; CP, crude protein; WSCs, water-soluble carbohydrates; NDF, neutral detergent fiber; ADF, acid detergent fiber; SEM, standard error of the mean; T, treatment; R, additive ratio; T*R, the interaction between the addition ratio and the type of tea residue added.

**Table 3:** The nitrogen components of tea residues with different addition ratios and types.

Treatment (T)	Ratio (R)	TN (% DM)	Composition of Total N (% TN)				
			TP-N	NPN	NH_3_-N	FAA-N	Peptide-N
CK	0%	0.938 ^h^	38.8 ^i^	61.2 ^c^	2.94 ^a^	20.8 ^a^	37.4 ^a^
G	5%	1.78 ^d^	63.9 ^de^	36.1 ^bc^	1.16 ^bcd^	5.79 ^e^	29.13 ^c^
	10%	2.62 ^a^	68.6 ^ab^	31.4 ^ef^	1.03 ^bcd^	2.71 ^f^	27.68 ^c^
B	5%	1.71 ^d^	71.7 ^a^	28.3 ^f^	1.40 ^abc^	6.14 ^e^	20.7 ^d^
	10%	2.23 ^bc^	68.9 ^ab^	31.1 ^ef^	0.881 ^cd^	3.55 ^f^	26.6 ^c^
Z	5%	1.43 ^e^	55.7 ^f^	44.3 ^a^	1.13 ^bcd^	8.68 ^d^	34.5 ^ab^
	10%	2.15 ^c^	61.0 ^e^	39.0 ^b^	0.721 ^d^	3.90 ^f^	34.3 ^ab^
W	5%	1.11 ^g^	56.2 ^f^	43.8 ^a^	1.60 ^ab^	14.2 ^b^	27.9 ^c^
	10%	1.26 ^f^	64.8 ^cd^	35.2 ^cd^	1.40 ^abc^	11.3 ^c^	22.6 ^d^
D	5%	1.74 ^d^	62.0 ^de^	38.0 ^bc^	1.84 ^a^	6.35 ^e^	29.8 ^bc^
	10%	2.30 ^b^	67.4 ^bc^	32.6 ^de^	1.99 ^a^	3.59 ^f^	27.0 ^c^
SEM		0.0457	1.623	1.623	0.268	0.762	2.283
*p*-value	T	<0.001	<0.001	<0.001	<0.001	<0.001	<0.001
	R	0.073	<0.001	<0.001	0.038	<0.001	0.367
	T*R	<0.449	<0.001	<0.001	0.293	0.021	0.004

The different lowercase letters (a–i) above the column indicate significant differences among treatments (*p* < 0.05). CK, no addition; G, green tea; B, black tea; Z, raw Pu’er tea; W, white tea; D, ripe Pu’er tea; DM, dry matter; TN, total nitrogen; TP-N, true protein nitrogen; NPN, non-protein nitrogen; NH_3_-N, ammonia nitrogen; FAA-N, free amino acid nitrogen; Peptide-N, peptide nitrogen; SEM, standard error of the mean; T, treatment; R, additive ratio; T*R, the interaction between the addition ratio and the type of tea residue added.

**Table 4:** The effect of different types and ratios of tea residue on total gas and methane production after 72 h.

Treatments	Ratio	b (mL)	c (mL/h)	CH_4_ (mL)
CK		40.0 ^bc^	0.556 ^bc^	3.06 ^bc^
G	5%	50.5 ^a^	0.701 ^a^	4.96 ^a^
	10%	36.4 ^bc^	0.506 ^bc^	2.11 ^c^
B	5%	39.1 ^bc^	0.543 ^bc^	2.52 ^bc^
	10%	39.9 ^bc^	0.555 ^bc^	3.55 ^bc^
Z	5%	38.2 ^bc^	0.530 ^bc^	2.67 ^bc^
	10%	42.0 ^bc^	0.583 ^bc^	3.61 ^b^
W	5%	44.6 ^ab^	0.619 ^ab^	3.16 ^bc^
	10%	40.6 ^bc^	0.564 ^bc^	2.52 ^bc^
D	5%	42.4 ^bc^	0.589 ^bc^	3.07 ^bc^
	10%	34.2 ^c^	0.475 ^c^	2.79 ^bc^
SEM		2.533	0.035	0.438

The different lowercase letters (a–c) above the column indicate significant differences among treatments (*p* < 0.05). b, asymptotic gas production; c, the rate of fermentation. SEM, standard error of the mean.

## Data Availability

The raw sequences of the 16S rRNA gene were deposited in the NCBI database for public access (PRJNA1174398). All data are presented in the manuscript.

## Supplementary Materials

The following supporting information can be downloaded at https://www.mdpi.com/article/10.3390/microorganisms12112178/s1: Table S1: Fermentation characteristics of ensiled sweet sorghum treated with tea residues; Table S2: The microbial population of tea residue with different addition ratios and types; Table S3: Effects of adding different types and ratios of tea residue on the alpha diversity index of ensiled sweet sorghum; Figure S1: Principal coordinate analysis based on the bacterial diversity of ensiled sweet sorghum.
