# Supplementary material for: Effects of Different Types and Ratios of Dry Tea Residues on Nutrient Content, In Vitro Rumen Fermentation, and the Bacterial Community of Ensiled Sweet Sorghum
Source: Microorganisms. 2024 Oct 29;12(11):2178. doi: 10.3390/microorganisms12112178 (PMC11596653; doi:10.3390/microorganisms12112178)
Supplement: Supplementary file 1 [file microorganisms-12-02178-s001.zip › Supplementary material.pdf]

Table S1. Fermentation characteristics of ensiled sweet sorghum treated with tea residues.

Treatment (T)	Ratio (R)	pH	LA (g/kg DM)	AA (g/kg DM)	PA (g/kg DM)	BA (g/kg DM)
CK	0%	4.70[a]	27.0[cd]	3.90[c]	44.3[ab]	2.55[b]
G	5%	4.21[bc]	43.7[ab]	10.0[bc]	34.7[bc]	18.7[b]
	10%	4.72[a]	20.1[d]	16.2[b]	18.3[cd]	ND
B	5%	4.37[bc]	27.2[cd]	4.20[c]	22.4[cd]	ND
	10%	4.27[bc]	22.6[d]	5.23[c]	23.4[cd]	7.05[b]
Z	5%	4.12[c]	57.2[a]	7.73[bc]	30.0[bcd]	166[a]
	10%	4.22[bc]	37.3[bc]	4.83[c]	17.1[d]	194[a]
W	5%	4.16[bc]	53.5[a]	4.98[c]	31.3[bcd]	18.7[b]
	10%	4.22[bc]	51.3[a]	3.40[c]	23.7[cd]	17.1[b]
D	5%	4.46[ab]	58.0[a]	26.8[a]	58.0[a]	9.03[b]
	10%	4.68[a]	29.3[cd]	1.50[c]	34.0[bcd]	8.55[b]
SEM		0.102	0.473	0.340	0.508	2.086
	T	0.002	<0.001	0.007	<0.001	<0.001
P-value	R	0.007	<0.001	0.036	0.001	0.451
	T*R	0.081	0.020	<0.001	0.170	0.018

G, green tea; B, black tea; Z, raw Pu'er tea; W, white tea; D, ripe Pu'er tea; LA, lactic acid; AA, acetic acid; PA, propionic acid; BA, butyric acid; [a-d], means in the same column are different at a level of $P < 0.05$. SEM, standard error of the mean; T, treatments; R, additive ratio; T * R, the interaction between the addition ratio and the type of tea residue added.

Table S2. The microbial population of tea residue with different addition ratios and types.

Treatment (T)	Ratio (R)	LAB ($\log_{10}$ cfu g^{-1} FW)	EC ($\log_{10}$ cfu g^{-1} FW)	Yeasts ($\log_{10}$ cfu g^{-1} FW)	Moulds ($\log_{10}$ cfu g^{-1} FW)
CK	0%	5.64[bc]	ND	ND	ND
G	5%	5.65[bc]	ND	ND	ND
	10%	6.08[a]	ND	ND	ND
B	5%	5.38[c]	ND	ND	ND
	10%	5.65[bc]	ND	ND	ND
Z	5%	5.68[bc]	ND	ND	ND
	10%	5.48[c]	ND	ND	ND
W	5%	5.63[bc]	ND	ND	ND
	10%	5.95[ab]	ND	ND	ND
D	5%	6.10[a]	ND	ND	ND
	10%	6.20[a]	ND	ND	ND
SEM		0.118	ND	ND	ND
	T	<0.001	ND	ND	ND
P-value	R	0.019	ND	ND	ND
	T*R	0.097	ND	ND	ND

G, green tea; B, black tea; Z, raw Pu'er tea; W, white tea; D, ripe Pu'er tea; LAB, lactic acid bacteria; EC, escherichia coli. [a-c], means in the same column are different at a level of $P < 0.05$. ND, not detected; SEM, standard error of mean; T, treatments; R, additive ratio; T * R, the interaction between the addition ratio and the type of tea residue added.

Table S3. Effects of adding different types and ratios of tea residue on the alpha diversity index of ensiled sweet sorghum.

Treatment	Ratio	OTUs	Shannon	Simpson	Chao 1	Ace	Coverage
CK	0%	262	3.542	0.805	295	304	0.999
G	5%	205	2.717	0.666	247	265	0.999
	10%	178	2.067	0.557	234	244	0.999
B	5%	184	3.317	0.796	203	214	0.999
	10%	222	3.424	0.803	257	265	0.999
Z	5%	179	2.081	0.474	204	217	0.999
	10%	197	2.446	0.678	251	260	0.999
W	5%	187	2.634	0.644	206	218	0.999
	10%	184	2.143	0.564	215	227	0.999
D	5%	175	3.148	0.772	201	208	0.999
	10%	248	2.370	0.493	274	278	0.999

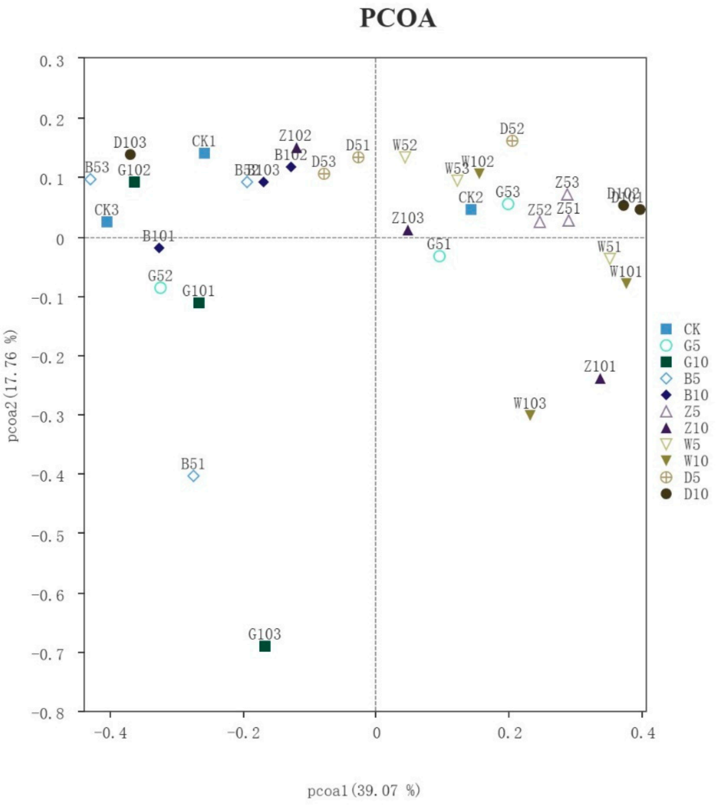

Figure S1. Principal coordinate analysis based on the bacterial diversity of ensiled sweet sorghum.
